# *catena*-Poly[4,4′-bipyridinium [[tetra­aqua­(pyridine-2,6-dicarboxyl­ato-κ^3^ *O* ^2^,*N*,*O* ^6^)cerate(III)]-μ-pyridine-2,6-dicarboxyl­ato-κ^4^ *O* ^2^:*O* ^2′^,*N*,*O* ^6^-[(pyridine-2,6-dicarboxyl­ato-κ^3^ *O* ^2^,*N*,*O* ^6^)cerate(III)]-μ-pyridine-2,6-dicarboxyl­ato-κ^4^ *O* ^2^,*N*,*O* ^6^:*O* ^6′^] penta­hydrate]

**DOI:** 10.1107/S1600536811004995

**Published:** 2011-02-12

**Authors:** Hossein Aghabozorg, Sara Omidvar, Masoud Mirzaei, Behrouz Notash

**Affiliations:** a Faculty of Chemistry, Islamic Azad University, North Tehran Branch, Tehran, Iran; b Department of Chemistry, School of Sciences, Ferdowsi University of Mashhad, Mashhad 917791436, Iran; c Department of Chemistry, Shahid Beheshti University, G. C., Evin, Tehran 1983963113, Iran

## Abstract

The title compound, {(C_10_H_10_N_2_)[Ce_2_(C_7_H_3_NO_4_)_4_(H_2_O)_4_]·5H_2_O}_*n*_, is composed of a one-dimensional anionic complex, a doubly protonated 4,4′-bipyridine mol­ecule as a counter-ion and five uncoordinated water mol­ecules. The anion bears two nine-coordinate Ce^III^ ions, each with a distorted tricapped trigonal–prismatic geometry. In the crystal, inter­molecular C—H⋯O, N—H⋯O and O—H⋯O hydrogen bonds, as well as π–π inter­actions with centroid–centroid distances of 3.514 (3) Å connect the various components into a supra­molecular structure.

## Related literature

For hydrogen-bonding inter­actions in proton-transfer compounds, see: Aghabozorg *et al.* (2008 ▶, 2010*a* ▶,*b* ▶); Sheshmani *et al.* (2005 ▶); Soleimannejad *et al.* (2007 ▶).
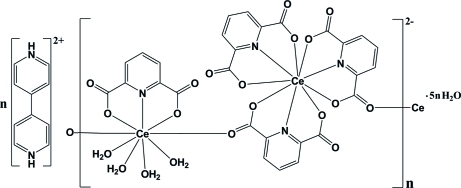


## Experimental

### 

#### Crystal data

(C_10_H_10_N_2_)[Ce_2_(C_7_H_3_NO_4_)_4_(H_2_O)_4_]·5H_2_O
*M* *_r_* = 1261
Triclinic, 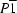

*a* = 13.010 (3) Å
*b* = 13.453 (3) Å
*c* = 13.586 (3) Å
α = 99.95 (3)°
β = 99.87 (3)°
γ = 104.58 (3)°
*V* = 2208.3 (10) Å^3^
*Z* = 2
Mo *K*α radiation
μ = 2.14 mm^−1^
*T* = 150 K
0.40 × 0.40 × 0.10 mm

#### Data collection

Stoe IPDS II diffractometer
Absorption correction: numerical (*X-SHAPE* and *X-RED32*; Stoe & Cie, 2005 ▶) *T*_min_ = 0.440, *T*_max_ = 0.805
25469 measured reflections
11857 independent reflections
10379 reflections with *I* > 2σ(*I*)
*R*_int_ = 0.078

#### Refinement

*R*[*F* ^2^ > 2σ(*F* ^2^)] = 0.045
*wR*(*F* ^2^) = 0.120
*S* = 1.05
11857 reflections
716 parameters
12 restraints
H atoms treated by a mixture of independent and constrained refinement
Δρ_max_ = 1.96 e Å^−3^
Δρ_min_ = −2.01 e Å^−3^

### 

Data collection: *X-AREA* (Stoe & Cie, 2005 ▶); cell refinement: *X-AREA*; data reduction: *X-AREA*; program(s) used to solve structure: *SHELXS97* (Sheldrick, 2008 ▶); program(s) used to refine structure: *SHELXL97* (Sheldrick, 2008 ▶); molecular graphics: *ORTEP-3* (Farrugia, 1997 ▶); software used to prepare material for publication: *WinGX* (Farrugia, 1999 ▶).

## Supplementary Material

Crystal structure: contains datablocks I, global. DOI: 10.1107/S1600536811004995/hy2405sup1.cif

Structure factors: contains datablocks I. DOI: 10.1107/S1600536811004995/hy2405Isup2.hkl

Additional supplementary materials:  crystallographic information; 3D view; checkCIF report

## Figures and Tables

**Table 1:** Hydrogen-bond geometry (Å, °)

*D*—H⋯*A*	*D*—H	H⋯*A*	*D*⋯*A*	*D*—H⋯*A*
N5—H5*A*⋯O14	0.82 (3)	1.83 (4)	2.647 (5)	175 (6)
N6—H6*A*⋯O11^i^	0.88 (4)	1.63 (4)	2.514 (5)	177 (8)
O17—H17*A*⋯O2	0.82 (7)	2.05 (7)	2.866 (4)	175 (6)
O17—H17*B*⋯O13^ii^	0.83 (8)	1.88 (8)	2.681 (4)	161 (7)
O18—H18*A*⋯O1	0.88 (4)	2.15 (5)	2.983 (5)	157 (7)
O18—H18*B*⋯O16^iii^	0.90 (4)	1.95 (4)	2.837 (4)	171 (6)
O19—H19*A*⋯O15^iii^	0.82 (4)	1.93 (4)	2.736 (4)	167 (10)
O19—H19*B*⋯O21^iv^	0.88 (9)	1.81 (9)	2.689 (5)	171 (8)
O20—H20*A*⋯O22	0.85 (8)	2.17 (8)	2.894 (6)	142 (7)
O20—H20*B*⋯O23^v^	0.73 (10)	2.15 (10)	2.847 (5)	158 (10)
O21—H21*A*⋯O1^ii^	0.89 (4)	1.86 (4)	2.736 (5)	169 (6)
O21—H21*B*⋯O6^vi^	0.93 (9)	1.93 (9)	2.851 (5)	171 (8)
O22—H22*A*⋯O6^vii^	0.89 (8)	2.49 (8)	3.127 (5)	128 (6)
O22—H22*A*⋯O10^vii^	0.89 (8)	2.35 (8)	3.116 (5)	143 (7)
O22—H22*B*⋯O23^iv^	0.84 (4)	2.12 (7)	2.884 (7)	150 (6)
O23—H23*A*⋯O5^vi^	0.76 (10)	1.99 (10)	2.736 (6)	170 (12)
O23—H23*B*⋯O24^v^	0.71 (9)	2.30 (11)	2.926 (6)	149 (16)
O24—H24*A*⋯O15^i^	0.87 (15)	2.02 (15)	2.882 (5)	170 (15)
O24—H24*B*⋯O22	0.9 (2)	1.9 (2)	2.764 (6)	161
O25—H25*A*⋯O24	0.92 (11)	2.52 (11)	3.137 (7)	124 (10)
O25—H25*B*⋯O1^i^	0.91 (10)	2.43 (12)	3.120 (7)	133 (12)
O25—H25*B*⋯O17^i^	0.91 (10)	2.32 (13)	3.034 (7)	136 (15)
C5—H5⋯O16^viii^	0.93	2.46	3.380 (5)	170
C11—H11⋯O9^ix^	0.93	2.40	3.193 (6)	143
C24—H24⋯O8^ii^	0.93	2.45	3.172 (5)	135
C29—H29⋯O5^ix^	0.93	2.54	3.350 (6)	146
C30—H30⋯O7	0.93	2.28	3.001 (5)	134
C31—H31⋯O25^v^	0.93	2.45	3.225 (8)	141
C36—H36⋯O4^x^	0.93	2.34	3.178 (5)	150

Symmetry codes: (i) 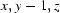; (ii) 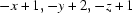; (iii) 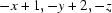; (iv) 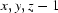; (v) 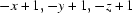; (vi) 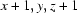; (vii) 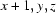; (viii) 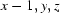; (ix) 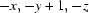; (x) 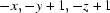.

**Fig. 1.**
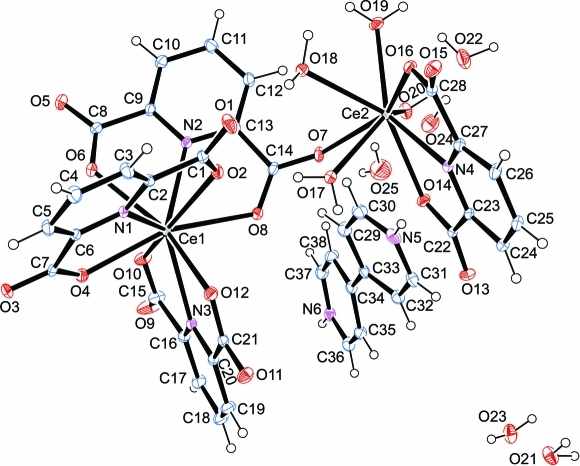
The asymmetric unit of the title compound, with displacement ellipsoids drawn at the 50% probability level.

**Fig. 2.**
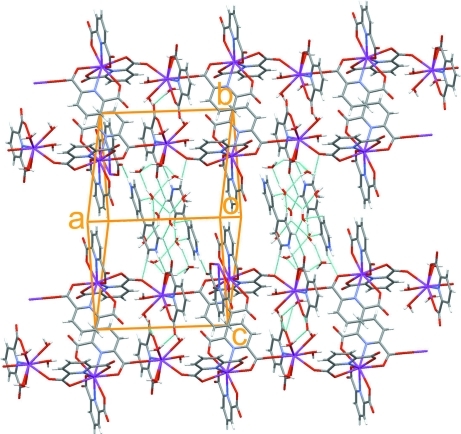
The packing diagram of the title compound, showing polymeric structure. The intermolecular C—H···O, N—H···O and O—H···O hydrogen bonds are shown as dashed lines.

**Fig. 3.**
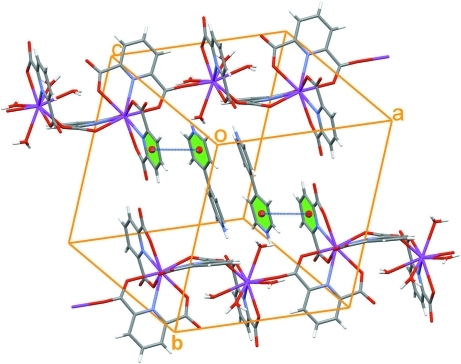
The packing diagram of the title compound, showing intermolecular π–π interactions (dashed lines) between pyridine rings of pydc ligands and bipy groups. Uncoordinated water molecules have been omitted for clarity.

## supplementary crystallographic information

### Comment

Through crystal engineering of some classes of crystalline compounds, one can conclude that inter- and/or intramolecular interctions such as hydrogen bonding, π–π stacking, ion pairing, and donor-acceptor interactions are famous for making aggregates of molecules. One or more of these interactions may result in the formation of specific and spontaneous self-associated compounds. Previous researches have shown that hydrogen bonding plays a key role in the preparation of self-assembled compounds. There is a very close relationship between hydrogen bonding and the formation of proton transfer compounds (Aghabozorg *et al.*, 2008, 2010*a*,b; Sheshmani *et al.*, 2005; Soleimannejad *et al.*, 2007).

Herein, we report the synthesis and crystal structure of the title compound. The compound is composed of a one-dimensional anionic complex, [Ce_2_(pydc)_4_(H_2_O)_4_]^2-^ (pydc = pyridine-2,6-dicarboxylate), a protonated 4,4'-bipyridine, (bipyH_2_)^2+^, as a counterion, and five uncoordinated water molecules (Fig. 1). The Ce1 atom is nine-coordinated by three pydc ligands, which act as tridentate ligands through two O atoms and one N atom. The Ce2 atom is nine-coordinated by one tridentate pydc ligand, four O atoms of coordinated water molecules and two O atoms from the carboxylate groups of the Ce1 coordination environment. By considering the angles between the atoms of coordination environment, the geometry around Ce atoms can be described as highly distorted tricapped trigonal-prismatic. The title compound shows a one-dimensional polymeric structure (Fig. 2). An important feature of the compound is the presence of extensive intermolecular O—H···O, N—H···O and C—H···O hydrogen bonds (Table 1, Fig. 2), which seem to be effective in the stabilization of the crystal structure. There is also π–π interaction, with centroid–centroid distance of 3.514 (3) Å between the pyridine ring of pydc and bipyridine (Fig. 3). This non-covalent interaction results in the formation of an interesting supramolecular structure.

### Experimental

A solution of Ce(NO_3_)_3_ (109 mg, 0.25 mmol) in water (10 ml) was added to an aqueous solution of pyridine-2,6-dicarboxylic acid (85 mg, 0.50 mmol) and 4,4'-bipyridine (78 mg, 0.50 mmol) in water (35 ml) in a 1:2:2 molar ratio and refluxed for an hour. Plate yellow crystals of the title compound were obtained by allowing the mixture to stand at room temperature (yield: 40.5%; m. p.: 270°C).

### Refinement

H atoms of the protonated nitrogen of bipyridine and water molecules were found in a diference Fourier map and refined isotropically, except H24A, H24B, H25A and H25B which were refined with *U*_iso_(H) = 1.5*U*_eq_(O). The H atoms of the protonated nitrogen and water molecules, H5A, H6A, H18A, H18B, H19A, H21A, H22B, H23B, H25A and H25B were refined with distance restraints of N—H/O—H = 0.82 (3), 0.88 (4), 0.88 (4), 0.90 (4), 0.82 (4), 0.89 (4), 0.84 (4), 0.71 (9), 0.92 (11), 0.91 (10) Å, respectively. Also H···H distance restraints of 1.3 (4) and 1.6 (4) Å for H23A···H23B and H25A···H25B, respectively, were used. H atoms on C atoms were positioned geometrically and refined as riding atoms, with C—H = 0.93 Å and *U*_iso_(H) = 1.2*U*_eq_(C). The highest residual electron density was found at 1.24 Å from Ce2 atom and the deepest hole at 0.55 Å from Ce2 atom.

### Crystal data

**Table:**

(C_10_H_10_N_2_)[Ce_2_(C_7_H_3_NO_4_)_4_(H_2_O)_4_]·5H_2_O	*Z* = 2
*M**_r_* = 1261	*F*(000) = 1252
Triclinic, *P*1	*D*_x_ = 1.896 Mg m^−^^3^
Hall symbol: -P 1	Mo *K*α radiation, λ = 0.71073 Å
*a* = 13.010 (3) Å	Cell parameters from 11857 reflections
*b* = 13.453 (3) Å	θ = 2.5–29.1°
*c* = 13.586 (3) Å	µ = 2.14 mm^−^^1^
α = 99.95 (3)°	*T* = 150 K
β = 99.87 (3)°	Plate, yellow
γ = 104.58 (3)°	0.40 × 0.40 × 0.10 mm
*V* = 2208.3 (10) Å^3^	

### Data collection

**Table:**

Stoe IPDS II diffractometer	11857 independent reflections
Radiation source: fine-focus sealed tube	10379 reflections with *I* > 2σ(*I*)
graphite	*R*_int_ = 0.078
ω scans	θ_max_ = 29.1°, θ_min_ = 2.5°
Absorption correction: numerical (*X-SHAPE* and *X-RED32*; Stoe & Cie, 2005)	*h* = −15→17
*T*_min_ = 0.440, *T*_max_ = 0.805	*k* = −18→18
25469 measured reflections	*l* = −18→18

### Refinement

**Table:**

Refinement on *F*^2^	Primary atom site location: structure-invariant direct methods
Least-squares matrix: full	Secondary atom site location: difference Fourier map
*R*[*F*^2^ > 2σ(*F*^2^)] = 0.045	Hydrogen site location: inferred from neighbouring sites
*wR*(*F*^2^) = 0.120	H atoms treated by a mixture of independent and constrained refinement
*S* = 1.05	*w* = 1/[σ^2^(*F*_o_^2^) + (0.064*P*)^2^ + 5.1288*P*] where *P* = (*F*_o_^2^ + 2*F*_c_^2^)/3
11857 reflections	(Δ/σ)_max_ = 0.001
716 parameters	Δρ_max_ = 1.96 e Å^−^^3^
12 restraints	Δρ_min_ = −2.01 e Å^−^^3^

### Fractional atomic coordinates and isotropic or equivalent isotropic displacement parameters (Å^2^)

**Table:**

	*x*	*y*	*z*	*U*_iso_*/*U*_eq_	
O23	0.6037 (3)	0.5090 (3)	0.9068 (4)	0.0387 (9)	
Ce1	0.003047 (16)	0.783224 (16)	0.218322 (15)	0.01548 (7)	
Ce2	0.503761 (15)	0.888641 (16)	0.188431 (15)	0.01464 (7)	
O1	0.2143 (3)	1.0504 (3)	0.1004 (3)	0.0322 (8)	
O2	0.1494 (2)	0.9110 (2)	0.1619 (2)	0.0218 (6)	
O3	−0.3036 (2)	0.8972 (3)	0.2097 (2)	0.0224 (6)	
O4	−0.1754 (2)	0.8143 (2)	0.2351 (2)	0.0209 (6)	
O5	−0.1869 (3)	0.5884 (3)	−0.1116 (2)	0.0318 (7)	
O6	−0.1349 (2)	0.6945 (3)	0.0453 (2)	0.0231 (6)	
O7	0.3383 (2)	0.7579 (2)	0.2004 (2)	0.0219 (6)	
O8	0.1860 (2)	0.7463 (2)	0.2582 (2)	0.0206 (6)	
O9	−0.1245 (4)	0.4852 (3)	0.3346 (3)	0.0468 (11)	
O10	−0.0903 (3)	0.6100 (2)	0.2430 (2)	0.0258 (6)	
O11	0.1350 (3)	1.0213 (2)	0.5401 (2)	0.0252 (6)	
O12	0.0911 (2)	0.9458 (2)	0.3715 (2)	0.0220 (6)	
O13	0.6021 (3)	0.9301 (3)	0.5394 (2)	0.0246 (6)	
O14	0.5409 (2)	0.8720 (2)	0.3707 (2)	0.0214 (6)	
O15	0.6315 (3)	1.2353 (2)	0.1697 (2)	0.0267 (7)	
O16	0.5796 (2)	1.0588 (2)	0.1370 (2)	0.0188 (5)	
O17	0.3761 (3)	0.9864 (3)	0.2614 (2)	0.0236 (6)	
O18	0.3581 (2)	0.9170 (3)	0.0496 (2)	0.0231 (6)	
O19	0.5152 (3)	0.8104 (3)	0.0138 (2)	0.0307 (8)	
O20	0.5305 (3)	0.7001 (3)	0.1861 (3)	0.0252 (6)	
O21	0.6923 (3)	0.7702 (3)	0.9590 (3)	0.0336 (8)	
O22	0.6705 (3)	0.5749 (3)	0.1273 (4)	0.0424 (9)	
O24	0.5617 (4)	0.4218 (3)	0.2152 (3)	0.0414 (9)	
O25	0.4130 (5)	0.2170 (5)	0.2555 (4)	0.0704 (15)	
N1	−0.0380 (3)	0.9504 (3)	0.1641 (2)	0.0166 (6)	
N2	0.0640 (3)	0.6775 (3)	0.0686 (3)	0.0176 (6)	
N3	0.0062 (3)	0.7581 (3)	0.4076 (3)	0.0168 (6)	
N4	0.6135 (3)	1.0587 (3)	0.3339 (2)	0.0175 (6)	
N5	0.4495 (3)	0.6883 (3)	0.4101 (3)	0.0284 (8)	
N6	0.2235 (3)	0.1985 (3)	0.5090 (3)	0.0248 (7)	
C1	0.1421 (3)	0.9929 (3)	0.1318 (3)	0.0187 (7)	
C2	0.0364 (3)	1.0209 (3)	0.1351 (3)	0.0175 (7)	
C3	0.0183 (3)	1.1118 (4)	0.1106 (3)	0.0241 (8)	
H3	0.0721	1.1607	0.0926	0.029*	
C4	−0.0831 (4)	1.1277 (4)	0.1137 (4)	0.0257 (9)	
H4	−0.0986	1.1868	0.0957	0.031*	
C5	−0.1604 (3)	1.0556 (3)	0.1437 (3)	0.0224 (8)	
H5	−0.2280	1.0655	0.1471	0.027*	
C6	−0.1341 (3)	0.9675 (3)	0.1689 (3)	0.0159 (7)	
C7	−0.2116 (3)	0.8863 (3)	0.2073 (3)	0.0171 (7)	
C8	−0.1185 (3)	0.6374 (3)	−0.0315 (3)	0.0202 (8)	
C9	−0.0023 (3)	0.6335 (3)	−0.0242 (3)	0.0183 (7)	
C10	0.0355 (3)	0.5935 (4)	−0.1072 (3)	0.0232 (8)	
H10	−0.0128	0.5599	−0.1698	0.028*	
C11	0.1463 (4)	0.6042 (4)	−0.0960 (4)	0.0258 (9)	
H11	0.1739	0.5810	−0.1517	0.031*	
C12	0.2147 (3)	0.6503 (3)	0.0002 (3)	0.0222 (8)	
H12	0.2892	0.6583	0.0105	0.027*	
C13	0.1700 (3)	0.6841 (3)	0.0808 (3)	0.0184 (7)	
C14	0.2363 (3)	0.7337 (3)	0.1887 (3)	0.0186 (7)	
C15	−0.0873 (4)	0.5764 (3)	0.3261 (3)	0.0269 (9)	
C16	−0.0324 (3)	0.6614 (3)	0.4231 (3)	0.0201 (8)	
C17	−0.0270 (4)	0.6413 (3)	0.5207 (3)	0.0235 (8)	
H17	−0.0523	0.5730	0.5293	0.028*	
C18	0.0169 (4)	0.7257 (4)	0.6047 (3)	0.0265 (9)	
H18	0.0215	0.7147	0.6709	0.032*	
C19	0.0540 (4)	0.8269 (4)	0.5891 (3)	0.0239 (8)	
H19	0.0817	0.8850	0.6442	0.029*	
C20	0.0486 (3)	0.8388 (3)	0.4890 (3)	0.0187 (7)	
C21	0.0944 (3)	0.9433 (3)	0.4635 (3)	0.0182 (7)	
C22	0.5907 (3)	0.9444 (3)	0.4513 (3)	0.0170 (7)	
C23	0.6353 (3)	1.0531 (3)	0.4325 (3)	0.0167 (7)	
C24	0.6926 (3)	1.1406 (3)	0.5114 (3)	0.0210 (8)	
H24	0.7049	1.1351	0.5795	0.025*	
C25	0.7310 (3)	1.2366 (4)	0.4853 (3)	0.0231 (8)	
H25	0.7712	1.2961	0.5360	0.028*	
C26	0.7091 (3)	1.2435 (3)	0.3829 (3)	0.0218 (8)	
H26	0.7342	1.3069	0.3641	0.026*	
C27	0.6485 (3)	1.1525 (3)	0.3101 (3)	0.0178 (7)	
C28	0.6175 (3)	1.1501 (3)	0.1968 (3)	0.0193 (7)	
C29	0.3327 (4)	0.5148 (4)	0.3526 (4)	0.0292 (9)	
H29	0.2852	0.4613	0.2995	0.035*	
C30	0.3824 (4)	0.6105 (4)	0.3339 (4)	0.0301 (10)	
H30	0.3691	0.6211	0.2676	0.036*	
C31	0.4700 (3)	0.6775 (4)	0.5069 (4)	0.0251 (9)	
H31	0.5158	0.7336	0.5586	0.030*	
C32	0.4232 (4)	0.5828 (3)	0.5297 (3)	0.0245 (8)	
H32	0.4375	0.5750	0.5969	0.029*	
C33	0.3541 (3)	0.4985 (3)	0.4518 (3)	0.0214 (8)	
C34	0.3074 (3)	0.3929 (3)	0.4717 (3)	0.0223 (8)	
C35	0.3043 (3)	0.3822 (3)	0.5715 (3)	0.0237 (8)	
H35	0.3303	0.4405	0.6264	0.028*	
C36	0.2614 (3)	0.2823 (4)	0.5865 (4)	0.0254 (9)	
H36	0.2592	0.2738	0.6527	0.030*	
C37	0.2270 (4)	0.2074 (4)	0.4136 (4)	0.0285 (9)	
H37	0.2010	0.1472	0.3606	0.034*	
C38	0.2682 (4)	0.3035 (4)	0.3914 (3)	0.0272 (9)	
H38	0.2699	0.3087	0.3243	0.033*	
H5A	0.476 (4)	0.743 (3)	0.394 (4)	0.031 (15)*	
H6A	0.193 (6)	0.137 (4)	0.522 (6)	0.07 (3)*	
H17A	0.311 (6)	0.965 (5)	0.236 (5)	0.040 (17)*	
H18A	0.333 (6)	0.971 (4)	0.070 (5)	0.06 (2)*	
H19A	0.468 (6)	0.804 (8)	−0.037 (5)	0.10 (3)*	
H20A	0.592 (6)	0.694 (6)	0.176 (5)	0.05 (2)*	
H21A	0.731 (5)	0.826 (4)	0.941 (5)	0.039 (17)*	
H22A	0.741 (7)	0.583 (6)	0.130 (6)	0.07 (2)*	
H23A	0.660 (8)	0.526 (9)	0.895 (9)	0.11 (4)*	
H24A	0.577 (12)	0.362 (12)	0.206 (11)	0.167*	
H25A	0.414 (11)	0.255 (9)	0.205 (8)	0.167*	
H17B	0.378 (6)	0.998 (6)	0.324 (6)	0.06 (2)*	
H18B	0.385 (5)	0.928 (5)	−0.005 (4)	0.048 (19)*	
H19B	0.575 (7)	0.795 (7)	0.002 (6)	0.08 (3)*	
H20B	0.483 (8)	0.654 (8)	0.163 (7)	0.09 (3)*	
H21B	0.743 (8)	0.739 (7)	0.985 (7)	0.08 (3)*	
H22B	0.633 (9)	0.566 (10)	0.068 (5)	0.14 (5)*	
H23B	0.560 (9)	0.532 (11)	0.898 (12)	0.17 (7)*	
H24B	0.609 (17)	0.464 (17)	0.184 (16)	0.260*	
H25B	0.365 (13)	0.152 (7)	0.243 (12)	0.260*	

### Atomic displacement parameters (Å^2^)

**Table:**

	*U*^11^	*U*^22^	*U*^33^	*U*^12^	*U*^13^	*U*^23^
O23	0.0246 (18)	0.034 (2)	0.052 (2)	0.0025 (15)	0.0044 (17)	0.0073 (17)
Ce1	0.01187 (10)	0.01963 (12)	0.01492 (11)	0.00337 (8)	0.00220 (8)	0.00646 (8)
Ce2	0.01032 (10)	0.02055 (12)	0.01249 (11)	0.00290 (8)	0.00114 (7)	0.00601 (8)
O1	0.0192 (14)	0.0407 (19)	0.047 (2)	0.0100 (13)	0.0144 (14)	0.0297 (17)
O2	0.0170 (13)	0.0260 (15)	0.0261 (15)	0.0073 (11)	0.0058 (11)	0.0130 (12)
O3	0.0129 (12)	0.0311 (16)	0.0252 (15)	0.0066 (11)	0.0073 (11)	0.0083 (12)
O4	0.0181 (13)	0.0255 (15)	0.0209 (14)	0.0047 (11)	0.0049 (11)	0.0119 (12)
O5	0.0185 (14)	0.046 (2)	0.0232 (16)	0.0043 (14)	−0.0029 (12)	0.0013 (14)
O6	0.0156 (13)	0.0306 (16)	0.0209 (14)	0.0061 (12)	−0.0002 (11)	0.0049 (12)
O7	0.0120 (12)	0.0260 (15)	0.0249 (15)	0.0030 (11)	−0.0011 (11)	0.0073 (12)
O8	0.0163 (13)	0.0297 (15)	0.0173 (13)	0.0083 (11)	0.0021 (10)	0.0084 (12)
O9	0.084 (3)	0.0209 (17)	0.0240 (17)	−0.0050 (18)	0.0098 (19)	0.0058 (14)
O10	0.0345 (17)	0.0192 (14)	0.0189 (14)	−0.0006 (12)	0.0046 (12)	0.0062 (11)
O11	0.0270 (15)	0.0212 (15)	0.0214 (14)	−0.0003 (12)	0.0004 (12)	0.0047 (12)
O12	0.0200 (14)	0.0241 (15)	0.0207 (14)	0.0036 (11)	0.0037 (11)	0.0066 (12)
O13	0.0293 (16)	0.0316 (16)	0.0143 (13)	0.0089 (13)	0.0050 (12)	0.0086 (12)
O14	0.0200 (13)	0.0250 (15)	0.0157 (13)	0.0000 (11)	0.0014 (11)	0.0075 (11)
O15	0.0314 (16)	0.0233 (15)	0.0231 (15)	0.0064 (13)	−0.0011 (13)	0.0088 (12)
O16	0.0183 (13)	0.0191 (13)	0.0159 (13)	0.0022 (11)	0.0007 (10)	0.0039 (11)
O17	0.0168 (14)	0.0355 (17)	0.0163 (14)	0.0072 (12)	0.0016 (11)	0.0025 (13)
O18	0.0190 (13)	0.0343 (17)	0.0188 (14)	0.0093 (12)	0.0038 (11)	0.0112 (13)
O19	0.0286 (17)	0.055 (2)	0.0164 (14)	0.0262 (16)	0.0035 (13)	0.0091 (14)
O20	0.0196 (15)	0.0231 (16)	0.0325 (17)	0.0052 (13)	0.0051 (13)	0.0078 (13)
O21	0.0273 (17)	0.0367 (19)	0.046 (2)	0.0141 (15)	0.0141 (15)	0.0209 (17)
O22	0.0213 (17)	0.044 (2)	0.054 (3)	0.0012 (16)	0.0097 (17)	0.0030 (19)
O24	0.047 (2)	0.038 (2)	0.038 (2)	0.0150 (18)	0.0022 (17)	0.0082 (17)
O25	0.068 (4)	0.074 (4)	0.064 (3)	0.008 (3)	0.015 (3)	0.021 (3)
N1	0.0115 (14)	0.0223 (16)	0.0149 (14)	0.0036 (12)	0.0017 (11)	0.0048 (12)
N2	0.0146 (14)	0.0213 (16)	0.0161 (15)	0.0028 (12)	0.0020 (12)	0.0073 (13)
N3	0.0135 (14)	0.0186 (15)	0.0182 (15)	0.0038 (12)	0.0030 (12)	0.0059 (12)
N4	0.0130 (14)	0.0234 (17)	0.0157 (15)	0.0057 (12)	0.0015 (12)	0.0044 (13)
N5	0.0245 (18)	0.028 (2)	0.040 (2)	0.0082 (15)	0.0157 (16)	0.0189 (18)
N6	0.0173 (16)	0.0251 (18)	0.031 (2)	0.0022 (14)	0.0051 (14)	0.0114 (16)
C1	0.0146 (16)	0.0237 (19)	0.0186 (18)	0.0049 (14)	0.0038 (14)	0.0075 (15)
C2	0.0130 (16)	0.0236 (19)	0.0162 (17)	0.0011 (14)	0.0038 (13)	0.0107 (15)
C3	0.0203 (19)	0.028 (2)	0.030 (2)	0.0085 (16)	0.0095 (16)	0.0165 (18)
C4	0.025 (2)	0.027 (2)	0.030 (2)	0.0119 (17)	0.0060 (17)	0.0139 (18)
C5	0.0176 (18)	0.026 (2)	0.026 (2)	0.0073 (16)	0.0050 (15)	0.0108 (17)
C6	0.0136 (16)	0.0203 (18)	0.0143 (16)	0.0051 (13)	0.0017 (13)	0.0065 (14)
C7	0.0126 (16)	0.0205 (18)	0.0138 (16)	0.0013 (13)	−0.0017 (13)	0.0018 (14)
C8	0.0182 (18)	0.026 (2)	0.0151 (17)	0.0044 (15)	0.0018 (14)	0.0047 (15)
C9	0.0141 (16)	0.0205 (18)	0.0177 (17)	0.0026 (14)	0.0005 (14)	0.0040 (14)
C10	0.0203 (19)	0.027 (2)	0.0175 (18)	0.0029 (16)	0.0000 (15)	0.0024 (16)
C11	0.021 (2)	0.027 (2)	0.026 (2)	0.0049 (16)	0.0077 (16)	−0.0011 (17)
C12	0.0159 (17)	0.024 (2)	0.025 (2)	0.0042 (15)	0.0030 (15)	0.0034 (16)
C13	0.0140 (16)	0.0209 (18)	0.0193 (18)	0.0046 (14)	0.0012 (14)	0.0053 (15)
C14	0.0154 (17)	0.0209 (18)	0.0181 (18)	0.0030 (14)	−0.0006 (14)	0.0088 (15)
C15	0.033 (2)	0.021 (2)	0.023 (2)	0.0001 (17)	0.0067 (17)	0.0047 (16)
C16	0.0177 (17)	0.0240 (19)	0.0195 (18)	0.0051 (15)	0.0046 (14)	0.0086 (15)
C17	0.026 (2)	0.025 (2)	0.023 (2)	0.0084 (16)	0.0052 (16)	0.0125 (17)
C18	0.028 (2)	0.030 (2)	0.0191 (19)	0.0042 (17)	0.0019 (16)	0.0084 (17)
C19	0.026 (2)	0.027 (2)	0.0160 (18)	0.0033 (16)	0.0027 (15)	0.0049 (16)
C20	0.0138 (16)	0.0258 (19)	0.0175 (17)	0.0054 (14)	0.0032 (14)	0.0087 (15)
C21	0.0125 (16)	0.0238 (19)	0.0181 (17)	0.0034 (14)	0.0037 (13)	0.0071 (15)
C22	0.0124 (15)	0.0259 (19)	0.0128 (16)	0.0062 (14)	0.0017 (13)	0.0051 (14)
C23	0.0121 (15)	0.0251 (19)	0.0135 (16)	0.0083 (14)	0.0011 (13)	0.0031 (14)
C24	0.0179 (17)	0.028 (2)	0.0163 (17)	0.0064 (15)	0.0015 (14)	0.0044 (16)
C25	0.0149 (17)	0.030 (2)	0.0202 (19)	0.0046 (15)	−0.0013 (14)	0.0014 (16)
C26	0.0171 (17)	0.0203 (19)	0.0226 (19)	0.0001 (15)	−0.0019 (15)	0.0051 (16)
C27	0.0150 (16)	0.0197 (18)	0.0176 (17)	0.0049 (14)	0.0021 (13)	0.0031 (14)
C28	0.0126 (16)	0.027 (2)	0.0182 (18)	0.0039 (14)	0.0017 (14)	0.0080 (15)
C29	0.026 (2)	0.032 (2)	0.030 (2)	0.0034 (18)	0.0062 (18)	0.0158 (19)
C30	0.031 (2)	0.036 (3)	0.031 (2)	0.012 (2)	0.0109 (19)	0.017 (2)
C31	0.0186 (18)	0.025 (2)	0.034 (2)	0.0056 (16)	0.0092 (17)	0.0117 (18)
C32	0.023 (2)	0.026 (2)	0.027 (2)	0.0081 (17)	0.0070 (16)	0.0098 (17)
C33	0.0157 (17)	0.026 (2)	0.027 (2)	0.0058 (15)	0.0070 (15)	0.0140 (17)
C34	0.0181 (18)	0.0221 (19)	0.025 (2)	0.0014 (15)	0.0043 (15)	0.0097 (16)
C35	0.0215 (19)	0.024 (2)	0.025 (2)	0.0023 (16)	0.0054 (16)	0.0097 (16)
C36	0.023 (2)	0.026 (2)	0.030 (2)	0.0047 (16)	0.0060 (17)	0.0153 (18)
C37	0.026 (2)	0.025 (2)	0.031 (2)	0.0041 (17)	0.0027 (18)	0.0075 (18)
C38	0.028 (2)	0.029 (2)	0.023 (2)	0.0033 (17)	0.0037 (17)	0.0086 (17)

### Geometric parameters (Å, °)

**Table:**

O23—H23A	0.76 (10)	N4—C27	1.345 (5)
O23—H23B	0.71 (9)	N5—C30	1.336 (7)
Ce1—O10	2.450 (3)	N5—C31	1.337 (6)
Ce1—O4	2.500 (3)	N5—H5A	0.82 (3)
Ce1—O8	2.540 (3)	N6—C36	1.325 (6)
Ce1—O2	2.545 (3)	N6—C37	1.330 (6)
Ce1—O6	2.584 (3)	N6—H6A	0.88 (4)
Ce1—O12	2.603 (3)	C1—C2	1.520 (5)
Ce1—N2	2.629 (4)	C2—C3	1.385 (6)
Ce1—N1	2.631 (3)	C3—C4	1.396 (6)
Ce1—N3	2.646 (3)	C3—H3	0.9300
Ce2—O3^i^	2.444 (3)	C4—C5	1.380 (6)
Ce2—O7	2.464 (3)	C4—H4	0.9300
Ce2—O19	2.469 (3)	C5—C6	1.393 (5)
Ce2—O14	2.501 (3)	C5—H5	0.9300
Ce2—O16	2.520 (3)	C6—C7	1.519 (5)
Ce2—O18	2.585 (3)	C8—C9	1.512 (5)
Ce2—O17	2.585 (3)	C9—C10	1.384 (6)
Ce2—O20	2.641 (3)	C10—C11	1.391 (6)
Ce2—N4	2.646 (4)	C10—H10	0.9300
O1—C1	1.244 (5)	C11—C12	1.390 (6)
O2—C1	1.259 (5)	C11—H11	0.9300
O3—C7	1.247 (5)	C12—C13	1.387 (6)
O3—Ce2^ii^	2.444 (3)	C12—H12	0.9300
O4—C7	1.265 (5)	C13—C14	1.509 (5)
O5—C8	1.239 (5)	C15—C16	1.517 (6)
O6—C8	1.267 (5)	C16—C17	1.392 (6)
O7—C14	1.258 (5)	C17—C18	1.385 (6)
O8—C14	1.250 (5)	C17—H17	0.9300
O9—C15	1.233 (5)	C18—C19	1.392 (6)
O10—C15	1.285 (5)	C18—H18	0.9300
O11—C21	1.273 (5)	C19—C20	1.389 (5)
O12—C21	1.250 (5)	C19—H19	0.9300
O13—C22	1.236 (5)	C20—C21	1.507 (5)
O14—C22	1.279 (5)	C22—C23	1.514 (6)
O15—C28	1.243 (5)	C23—C24	1.393 (6)
O16—C28	1.275 (5)	C24—C25	1.392 (6)
O17—H17A	0.82 (7)	C24—H24	0.9300
O17—H17B	0.83 (8)	C25—C26	1.395 (6)
O18—H18B	0.90 (4)	C25—H25	0.9300
O18—H18A	0.88 (4)	C26—C27	1.388 (6)
O18—H18B	0.90 (4)	C26—H26	0.9300
O19—H19A	0.82 (4)	C27—C28	1.515 (5)
O19—H19B	0.88 (9)	C29—C30	1.374 (6)
O20—H20A	0.85 (8)	C29—C33	1.395 (6)
O20—H20B	0.73 (10)	C29—H29	0.9300
O21—H21A	0.89 (4)	C30—H30	0.9300
O21—H21B	0.93 (9)	C31—C32	1.378 (6)
O22—H22A	0.89 (8)	C31—H31	0.9300
O22—H22B	0.84 (4)	C32—C33	1.401 (6)
O24—H24A	0.87 (15)	C32—H32	0.9300
O24—H24B	0.9 (2)	C33—C34	1.488 (6)
O25—H25A	0.92 (11)	C34—C38	1.394 (6)
O25—H25B	0.91 (10)	C34—C35	1.394 (6)
N1—C6	1.338 (5)	C35—C36	1.384 (6)
N1—C2	1.338 (5)	C35—H35	0.9300
N2—C13	1.339 (5)	C36—H36	0.9300
N2—C9	1.339 (5)	C37—C38	1.375 (6)
N3—C20	1.334 (5)	C37—H37	0.9300
N3—C16	1.338 (5)	C38—H38	0.9300
N4—C23	1.340 (5)		
			
H23A—O23—H23B	129 (10)	C36—N6—H6A	118 (5)
O10—Ce1—O4	81.20 (11)	C37—N6—H6A	121 (5)
O10—Ce1—O8	90.78 (11)	O1—C1—O2	124.6 (4)
O4—Ce1—O8	162.20 (9)	O1—C1—C2	119.4 (4)
O10—Ce1—O2	154.12 (11)	O2—C1—C2	116.1 (3)
O4—Ce1—O2	123.00 (9)	N1—C2—C3	122.6 (4)
O8—Ce1—O2	68.38 (9)	N1—C2—C1	114.3 (3)
O10—Ce1—O6	76.37 (10)	C3—C2—C1	123.1 (3)
O4—Ce1—O6	72.21 (10)	C2—C3—C4	118.0 (4)
O8—Ce1—O6	121.45 (10)	C2—C3—H3	121.0
O2—Ce1—O6	100.91 (10)	C4—C3—H3	121.0
O10—Ce1—O12	121.94 (10)	C5—C4—C3	119.9 (4)
O4—Ce1—O12	86.20 (10)	C5—C4—H4	120.1
O8—Ce1—O12	84.62 (10)	C3—C4—H4	120.1
O2—Ce1—O12	72.93 (10)	C4—C5—C6	118.1 (4)
O6—Ce1—O12	149.70 (10)	C4—C5—H5	121.0
O10—Ce1—N2	85.60 (11)	C6—C5—H5	121.0
O4—Ce1—N2	132.60 (10)	N1—C6—C5	122.5 (4)
O8—Ce1—N2	61.78 (10)	N1—C6—C7	115.5 (3)
O2—Ce1—N2	71.26 (10)	C5—C6—C7	122.0 (3)
O6—Ce1—N2	60.45 (10)	O3—C7—O4	126.2 (4)
O12—Ce1—N2	137.74 (10)	O3—C7—C6	117.6 (3)
O10—Ce1—N1	141.05 (11)	O4—C7—C6	116.1 (3)
O4—Ce1—N1	62.18 (10)	O5—C8—O6	126.4 (4)
O8—Ce1—N1	128.08 (10)	O5—C8—C9	118.1 (4)
O2—Ce1—N1	60.93 (10)	O6—C8—C9	115.5 (3)
O6—Ce1—N1	79.99 (10)	N2—C9—C10	121.9 (4)
O12—Ce1—N1	71.06 (10)	N2—C9—C8	114.6 (4)
N2—Ce1—N1	108.86 (10)	C10—C9—C8	123.4 (4)
O10—Ce1—N3	61.35 (11)	C9—C10—C11	119.4 (4)
O4—Ce1—N3	81.32 (10)	C9—C10—H10	120.3
O8—Ce1—N3	80.89 (10)	C11—C10—H10	120.3
O2—Ce1—N3	126.17 (10)	C12—C11—C10	118.4 (4)
O6—Ce1—N3	132.92 (10)	C12—C11—H11	120.8
O12—Ce1—N3	60.77 (10)	C10—C11—H11	120.8
N2—Ce1—N3	129.88 (10)	C13—C12—C11	118.8 (4)
N1—Ce1—N3	120.63 (10)	C13—C12—H12	120.6
O3^i^—Ce2—O7	134.99 (10)	C11—C12—H12	120.6
O3^i^—Ce2—O19	77.20 (11)	N2—C13—C12	122.5 (4)
O7—Ce2—O19	98.81 (12)	N2—C13—C14	114.3 (4)
O3^i^—Ce2—O14	81.51 (10)	C12—C13—C14	123.2 (3)
O7—Ce2—O14	75.82 (10)	O8—C14—O7	125.8 (4)
O19—Ce2—O14	143.15 (11)	O8—C14—C13	117.7 (3)
O3^i^—Ce2—O16	78.67 (10)	O7—C14—C13	116.4 (4)
O7—Ce2—O16	146.02 (9)	O9—C15—O10	127.5 (4)
O19—Ce2—O16	82.61 (12)	O9—C15—C16	118.2 (4)
O14—Ce2—O16	122.17 (10)	O10—C15—C16	114.3 (4)
O3^i^—Ce2—O18	135.63 (10)	N3—C16—C17	122.6 (4)
O7—Ce2—O18	78.75 (10)	N3—C16—C15	114.7 (3)
O19—Ce2—O18	68.25 (11)	C17—C16—C15	122.7 (4)
O14—Ce2—O18	142.09 (10)	C18—C17—C16	118.4 (4)
O16—Ce2—O18	70.25 (10)	C18—C17—H17	120.8
O3^i^—Ce2—O17	141.35 (11)	C16—C17—H17	120.8
O7—Ce2—O17	70.97 (11)	C17—C18—C19	119.3 (4)
O19—Ce2—O17	134.26 (10)	C17—C18—H18	120.3
O14—Ce2—O17	79.25 (10)	C19—C18—H18	120.3
O16—Ce2—O17	83.74 (10)	C20—C19—C18	118.2 (4)
O18—Ce2—O17	66.04 (10)	C20—C19—H19	120.9
O3^i^—Ce2—O20	69.27 (11)	C18—C19—H19	120.9
O7—Ce2—O20	67.13 (11)	N3—C20—C19	122.9 (4)
O19—Ce2—O20	71.40 (12)	N3—C20—C21	114.4 (3)
O14—Ce2—O20	73.06 (11)	C19—C20—C21	122.7 (4)
O16—Ce2—O20	142.18 (10)	O12—C21—O11	126.3 (4)
O18—Ce2—O20	121.16 (11)	O12—C21—C20	118.4 (4)
O17—Ce2—O20	134.03 (11)	O11—C21—C20	115.3 (3)
O3^i^—Ce2—N4	73.33 (11)	O13—C22—O14	124.2 (4)
O7—Ce2—N4	124.35 (11)	O13—C22—C23	120.6 (4)
O19—Ce2—N4	136.81 (12)	O14—C22—C23	115.2 (3)
O14—Ce2—N4	61.05 (10)	N4—C23—C24	122.7 (4)
O16—Ce2—N4	61.28 (10)	N4—C23—C22	114.5 (3)
O18—Ce2—N4	114.97 (11)	C24—C23—C22	122.8 (3)
O17—Ce2—N4	68.03 (10)	C25—C24—C23	117.9 (4)
O20—Ce2—N4	123.80 (11)	C25—C24—H24	121.0
C1—O2—Ce1	127.0 (2)	C23—C24—H24	121.0
C7—O3—Ce2^ii^	169.2 (3)	C24—C25—C26	120.0 (4)
C7—O4—Ce1	126.6 (2)	C24—C25—H25	120.0
C8—O6—Ce1	126.4 (2)	C26—C25—H25	120.0
C14—O7—Ce2	146.2 (3)	C27—C26—C25	117.8 (4)
C14—O8—Ce1	119.8 (2)	C27—C26—H26	121.1
C15—O10—Ce1	129.0 (3)	C25—C26—H26	121.1
C21—O12—Ce1	124.5 (3)	N4—C27—C26	122.9 (4)
C22—O14—Ce2	128.3 (2)	N4—C27—C28	114.2 (3)
C28—O16—Ce2	126.0 (2)	C26—C27—C28	122.9 (4)
Ce2—O17—H17A	119 (5)	O15—C28—O16	125.6 (4)
Ce2—O17—H17B	120 (5)	O15—C28—C27	118.5 (4)
H17A—O17—H17B	102 (7)	O16—C28—C27	115.9 (3)
H18B—O18—Ce2	110 (4)	C30—C29—C33	119.6 (5)
H18B—O18—H18A	107 (6)	C30—C29—H29	120.2
Ce2—O18—H18A	115 (5)	C33—C29—H29	120.2
Ce2—O18—H18B	110 (4)	N5—C30—C29	120.6 (4)
H18A—O18—H18B	107 (6)	N5—C30—H30	119.7
Ce2—O19—H19A	122 (7)	C29—C30—H30	119.7
Ce2—O19—H19B	121 (5)	N5—C31—C32	119.7 (4)
H19A—O19—H19B	116 (8)	N5—C31—H31	120.1
Ce2—O20—H20A	116 (5)	C32—C31—H31	120.1
Ce2—O20—H20B	119 (8)	C31—C32—C33	120.1 (4)
H20A—O20—H20B	115 (9)	C31—C32—H32	120.0
H21A—O21—H21B	105 (7)	C33—C32—H32	120.0
H22A—O22—H22B	114 (10)	C29—C33—C32	117.9 (4)
H24A—O24—H24B	104 (10)	C29—C33—C34	120.1 (4)
H25A—O25—H25B	120 (7)	C32—C33—C34	121.9 (4)
C6—N1—C2	118.9 (3)	C38—C34—C35	119.3 (4)
C6—N1—Ce1	119.4 (2)	C38—C34—C33	120.6 (4)
C2—N1—Ce1	121.7 (2)	C35—C34—C33	120.2 (4)
C13—N2—C9	118.9 (4)	C36—C35—C34	118.2 (4)
C13—N2—Ce1	117.7 (3)	C36—C35—H35	120.9
C9—N2—Ce1	122.4 (3)	C34—C35—H35	120.9
C20—N3—C16	118.6 (3)	N6—C36—C35	121.6 (4)
C20—N3—Ce1	121.9 (3)	N6—C36—H36	119.2
C16—N3—Ce1	119.5 (3)	C35—C36—H36	119.2
C23—N4—C27	118.7 (4)	N6—C37—C38	121.5 (5)
C23—N4—Ce2	120.8 (3)	N6—C37—H37	119.3
C27—N4—Ce2	120.5 (2)	C38—C37—H37	119.3
C30—N5—C31	122.0 (4)	C37—C38—C34	118.6 (4)
C30—N5—H5A	116 (4)	C37—C38—H38	120.7
C31—N5—H5A	122 (4)	C34—C38—H38	120.7
C36—N6—C37	120.9 (4)		
			
O10—Ce1—O2—C1	151.7 (3)	O3^i^—Ce2—N4—C27	90.7 (3)
O4—Ce1—O2—C1	−5.3 (4)	O7—Ce2—N4—C27	−135.7 (3)
O8—Ce1—O2—C1	−169.9 (4)	O19—Ce2—N4—C27	41.8 (3)
O6—Ce1—O2—C1	70.4 (3)	O14—Ce2—N4—C27	−179.9 (3)
O12—Ce1—O2—C1	−78.9 (3)	O16—Ce2—N4—C27	4.7 (3)
N2—Ce1—O2—C1	123.9 (3)	O18—Ce2—N4—C27	−42.4 (3)
N1—Ce1—O2—C1	−1.5 (3)	O17—Ce2—N4—C27	−90.3 (3)
N3—Ce1—O2—C1	−109.9 (3)	O20—Ce2—N4—C27	140.6 (3)
O10—Ce1—O4—C7	−161.8 (3)	Ce1—O2—C1—O1	−176.5 (3)
O8—Ce1—O4—C7	134.1 (3)	Ce1—O2—C1—C2	3.4 (5)
O2—Ce1—O4—C7	8.2 (3)	C6—N1—C2—C3	0.6 (6)
O6—Ce1—O4—C7	−83.4 (3)	Ce1—N1—C2—C3	−177.1 (3)
O12—Ce1—O4—C7	75.0 (3)	C6—N1—C2—C1	−179.7 (3)
N2—Ce1—O4—C7	−86.2 (3)	Ce1—N1—C2—C1	2.7 (4)
N1—Ce1—O4—C7	4.5 (3)	O1—C1—C2—N1	176.1 (4)
N3—Ce1—O4—C7	136.0 (3)	O2—C1—C2—N1	−3.8 (5)
O10—Ce1—O6—C8	−89.2 (3)	O1—C1—C2—C3	−4.2 (6)
O4—Ce1—O6—C8	−174.1 (3)	O2—C1—C2—C3	176.0 (4)
O8—Ce1—O6—C8	−6.7 (4)	N1—C2—C3—C4	−1.9 (7)
O2—Ce1—O6—C8	64.5 (3)	C1—C2—C3—C4	178.3 (4)
O12—Ce1—O6—C8	139.2 (3)	C2—C3—C4—C5	2.0 (7)
N2—Ce1—O6—C8	3.5 (3)	C3—C4—C5—C6	−0.8 (7)
N1—Ce1—O6—C8	122.0 (3)	C2—N1—C6—C5	0.7 (6)
N3—Ce1—O6—C8	−115.1 (3)	Ce1—N1—C6—C5	178.4 (3)
O3^i^—Ce2—O7—C14	−174.6 (5)	C2—N1—C6—C7	−177.2 (3)
O19—Ce2—O7—C14	−94.0 (5)	Ce1—N1—C6—C7	0.5 (4)
O14—Ce2—O7—C14	123.3 (5)	C4—C5—C6—N1	−0.6 (6)
O16—Ce2—O7—C14	−4.1 (6)	C4—C5—C6—C7	177.2 (4)
O18—Ce2—O7—C14	−28.4 (5)	Ce2^ii^—O3—C7—O4	−69.7 (16)
O17—Ce2—O7—C14	40.0 (5)	Ce2^ii^—O3—C7—C6	111.2 (14)
O20—Ce2—O7—C14	−159.4 (5)	Ce1—O4—C7—O3	175.0 (3)
N4—Ce2—O7—C14	84.3 (5)	Ce1—O4—C7—C6	−5.9 (5)
O10—Ce1—O8—C14	111.4 (3)	N1—C6—C7—O3	−177.6 (3)
O4—Ce1—O8—C14	174.1 (3)	C5—C6—C7—O3	4.4 (6)
O2—Ce1—O8—C14	−52.9 (3)	N1—C6—C7—O4	3.2 (5)
O6—Ce1—O8—C14	36.9 (3)	C5—C6—C7—O4	−174.8 (4)
O12—Ce1—O8—C14	−126.6 (3)	Ce1—O6—C8—O5	174.2 (3)
N2—Ce1—O8—C14	26.8 (3)	Ce1—O6—C8—C9	−8.4 (5)
N1—Ce1—O8—C14	−65.8 (3)	C13—N2—C9—C10	0.8 (6)
N3—Ce1—O8—C14	172.2 (3)	Ce1—N2—C9—C10	169.0 (3)
O4—Ce1—O10—C15	−95.0 (4)	C13—N2—C9—C8	−175.7 (3)
O8—Ce1—O10—C15	69.0 (4)	Ce1—N2—C9—C8	−7.5 (5)
O2—Ce1—O10—C15	104.3 (4)	O5—C8—C9—N2	−172.4 (4)
O6—Ce1—O10—C15	−168.7 (4)	O6—C8—C9—N2	10.0 (5)
O12—Ce1—O10—C15	−15.1 (4)	O5—C8—C9—C10	11.2 (6)
N2—Ce1—O10—C15	130.6 (4)	O6—C8—C9—C10	−166.5 (4)
N1—Ce1—O10—C15	−114.5 (4)	N2—C9—C10—C11	−3.6 (6)
N3—Ce1—O10—C15	−10.2 (4)	C8—C9—C10—C11	172.6 (4)
O10—Ce1—O12—C21	3.1 (3)	C9—C10—C11—C12	3.2 (7)
O4—Ce1—O12—C21	80.2 (3)	C10—C11—C12—C13	−0.3 (7)
O8—Ce1—O12—C21	−84.5 (3)	C9—N2—C13—C12	2.2 (6)
O2—Ce1—O12—C21	−153.5 (3)	Ce1—N2—C13—C12	−166.5 (3)
O6—Ce1—O12—C21	124.1 (3)	C9—N2—C13—C14	−179.2 (3)
N2—Ce1—O12—C21	−120.4 (3)	Ce1—N2—C13—C14	12.1 (4)
N1—Ce1—O12—C21	142.1 (3)	C11—C12—C13—N2	−2.5 (6)
N3—Ce1—O12—C21	−1.9 (3)	C11—C12—C13—C14	179.1 (4)
O3^i^—Ce2—O14—C22	76.6 (3)	Ce1—O8—C14—O7	148.6 (3)
O7—Ce2—O14—C22	−142.6 (3)	Ce1—O8—C14—C13	−32.1 (5)
O19—Ce2—O14—C22	131.7 (3)	Ce2—O7—C14—O8	−99.1 (5)
O16—Ce2—O14—C22	5.8 (4)	Ce2—O7—C14—C13	81.6 (6)
O18—Ce2—O14—C22	−93.3 (3)	N2—C13—C14—O8	12.3 (5)
O17—Ce2—O14—C22	−69.7 (3)	C12—C13—C14—O8	−169.2 (4)
O20—Ce2—O14—C22	147.4 (3)	N2—C13—C14—O7	−168.4 (3)
N4—Ce2—O14—C22	1.0 (3)	C12—C13—C14—O7	10.2 (6)
O3^i^—Ce2—O16—C28	−89.7 (3)	Ce1—O10—C15—O9	−170.5 (4)
O7—Ce2—O16—C28	97.1 (3)	Ce1—O10—C15—C16	10.4 (6)
O19—Ce2—O16—C28	−168.1 (3)	C20—N3—C16—C17	−1.9 (6)
O14—Ce2—O16—C28	−17.4 (3)	Ce1—N3—C16—C17	175.8 (3)
O18—Ce2—O16—C28	122.5 (3)	C20—N3—C16—C15	175.2 (4)
O17—Ce2—O16—C28	55.7 (3)	Ce1—N3—C16—C15	−7.1 (5)
O20—Ce2—O16—C28	−121.8 (3)	O9—C15—C16—N3	179.8 (5)
N4—Ce2—O16—C28	−12.6 (3)	O10—C15—C16—N3	−1.0 (6)
O3^i^—Ce2—O18—H18B	5(5)	O9—C15—C16—C17	−3.1 (7)
O7—Ce2—O18—H18B	−141 (5)	O10—C15—C16—C17	176.1 (4)
O19—Ce2—O18—H18B	−36 (5)	N3—C16—C17—C18	2.1 (6)
O14—Ce2—O18—H18B	171 (5)	C15—C16—C17—C18	−174.8 (4)
O16—Ce2—O18—H18B	53 (5)	C16—C17—C18—C19	0.0 (7)
O17—Ce2—O18—H18B	145 (5)	C17—C18—C19—C20	−2.0 (7)
O20—Ce2—O18—H18B	−86 (5)	C16—N3—C20—C19	−0.3 (6)
N4—Ce2—O18—H18B	96 (5)	Ce1—N3—C20—C19	−178.0 (3)
O10—Ce1—N1—C6	19.7 (4)	C16—N3—C20—C21	177.2 (3)
O4—Ce1—N1—C6	−2.2 (3)	Ce1—N3—C20—C21	−0.5 (4)
O8—Ce1—N1—C6	−164.8 (3)	C18—C19—C20—N3	2.3 (6)
O2—Ce1—N1—C6	−178.5 (3)	C18—C19—C20—C21	−175.1 (4)
O6—Ce1—N1—C6	72.9 (3)	Ce1—O12—C21—O11	−178.7 (3)
O12—Ce1—N1—C6	−98.0 (3)	Ce1—O12—C21—C20	2.5 (5)
N2—Ce1—N1—C6	126.8 (3)	N3—C20—C21—O12	−1.3 (5)
N3—Ce1—N1—C6	−61.4 (3)	C19—C20—C21—O12	176.3 (4)
O10—Ce1—N1—C2	−162.7 (3)	N3—C20—C21—O11	179.8 (3)
O4—Ce1—N1—C2	175.5 (3)	C19—C20—C21—O11	−2.6 (6)
O8—Ce1—N1—C2	12.9 (3)	Ce2—O14—C22—O13	177.2 (3)
O2—Ce1—N1—C2	−0.9 (3)	Ce2—O14—C22—C23	−1.9 (5)
O6—Ce1—N1—C2	−109.5 (3)	C27—N4—C23—C24	0.2 (6)
O12—Ce1—N1—C2	79.6 (3)	Ce2—N4—C23—C24	−179.9 (3)
N2—Ce1—N1—C2	−55.6 (3)	C27—N4—C23—C22	179.0 (3)
N3—Ce1—N1—C2	116.2 (3)	Ce2—N4—C23—C22	−1.1 (4)
O10—Ce1—N2—C13	−112.1 (3)	O13—C22—C23—N4	−177.4 (4)
O4—Ce1—N2—C13	174.1 (2)	O14—C22—C23—N4	1.8 (5)
O8—Ce1—N2—C13	−18.8 (3)	O13—C22—C23—C24	1.5 (6)
O2—Ce1—N2—C13	56.1 (3)	O14—C22—C23—C24	−179.3 (4)
O6—Ce1—N2—C13	171.1 (3)	N4—C23—C24—C25	−1.9 (6)
O12—Ce1—N2—C13	22.7 (3)	C22—C23—C24—C25	179.3 (4)
N1—Ce1—N2—C13	105.0 (3)	C23—C24—C25—C26	1.7 (6)
N3—Ce1—N2—C13	−65.8 (3)	C24—C25—C26—C27	0.2 (6)
O10—Ce1—N2—C9	79.6 (3)	C23—N4—C27—C26	1.9 (6)
O4—Ce1—N2—C9	5.8 (4)	Ce2—N4—C27—C26	−178.1 (3)
O8—Ce1—N2—C9	172.9 (3)	C23—N4—C27—C28	−178.9 (3)
O2—Ce1—N2—C9	−112.2 (3)	Ce2—N4—C27—C28	1.1 (4)
O6—Ce1—N2—C9	2.8 (3)	C25—C26—C27—N4	−2.0 (6)
O12—Ce1—N2—C9	−145.6 (3)	C25—C26—C27—C28	178.8 (4)
N1—Ce1—N2—C9	−63.3 (3)	Ce2—O16—C28—O15	−162.5 (3)
N3—Ce1—N2—C9	125.9 (3)	Ce2—O16—C28—C27	18.1 (5)
O10—Ce1—N3—C20	−174.1 (3)	N4—C27—C28—O15	169.0 (4)
O4—Ce1—N3—C20	−89.4 (3)	C26—C27—C28—O15	−11.8 (6)
O8—Ce1—N3—C20	90.0 (3)	N4—C27—C28—O16	−11.6 (5)
O2—Ce1—N3—C20	35.4 (3)	C26—C27—C28—O16	167.7 (4)
O6—Ce1—N3—C20	−145.1 (3)	C31—N5—C30—C29	−1.0 (7)
O12—Ce1—N3—C20	1.1 (3)	C33—C29—C30—N5	−1.0 (7)
N2—Ce1—N3—C20	130.7 (3)	C30—N5—C31—C32	1.6 (7)
N1—Ce1—N3—C20	−39.1 (3)	N5—C31—C32—C33	−0.2 (6)
O10—Ce1—N3—C16	8.3 (3)	C30—C29—C33—C32	2.3 (7)
O4—Ce1—N3—C16	92.9 (3)	C30—C29—C33—C34	−175.5 (4)
O8—Ce1—N3—C16	−87.7 (3)	C31—C32—C33—C29	−1.7 (6)
O2—Ce1—N3—C16	−142.2 (3)	C31—C32—C33—C34	176.0 (4)
O6—Ce1—N3—C16	37.3 (3)	C29—C33—C34—C38	17.7 (6)
O12—Ce1—N3—C16	−176.6 (3)	C32—C33—C34—C38	−160.0 (4)
N2—Ce1—N3—C16	−47.0 (3)	C29—C33—C34—C35	−163.5 (4)
N1—Ce1—N3—C16	143.2 (3)	C32—C33—C34—C35	18.8 (6)
O3^i^—Ce2—N4—C23	−89.2 (3)	C38—C34—C35—C36	−0.4 (6)
O7—Ce2—N4—C23	44.4 (3)	C33—C34—C35—C36	−179.2 (4)
O19—Ce2—N4—C23	−138.1 (3)	C37—N6—C36—C35	1.1 (7)
O14—Ce2—N4—C23	0.2 (3)	C34—C35—C36—N6	−0.4 (6)
O16—Ce2—N4—C23	−175.2 (3)	C36—N6—C37—C38	−1.1 (7)
O18—Ce2—N4—C23	137.7 (3)	N6—C37—C38—C34	0.3 (7)
O17—Ce2—N4—C23	89.8 (3)	C35—C34—C38—C37	0.4 (7)
O20—Ce2—N4—C23	−39.4 (3)	C33—C34—C38—C37	179.2 (4)

Symmetry codes: (i) *x*+1, *y*, *z*; (ii) *x*−1, *y*, *z*.

### Hydrogen-bond geometry (Å, °)

**Table:**

*D*—H···*A*	*D*—H	H···*A*	*D*···*A*	*D*—H···*A*
N5—H5A···O14	0.82 (3)	1.83 (4)	2.647 (5)	175 (6)
N6—H6A···O11^iii^	0.88 (4)	1.63 (4)	2.514 (5)	177 (8)
O17—H17A···O2	0.82 (7)	2.05 (7)	2.866 (4)	175 (6)
O17—H17B···O13^iv^	0.83 (8)	1.88 (8)	2.681 (4)	161 (7)
O18—H18A···O1	0.88 (4)	2.15 (5)	2.983 (5)	157 (7)
O18—H18B···O16^v^	0.90 (4)	1.95 (4)	2.837 (4)	171 (6)
O19—H19A···O15^v^	0.82 (4)	1.93 (4)	2.736 (4)	167 (10)
O19—H19B···O21^vi^	0.88 (9)	1.81 (9)	2.689 (5)	171 (8)
O20—H20A···O22	0.85 (8)	2.17 (8)	2.894 (6)	142 (7)
O20—H20B···O23^vii^	0.73 (10)	2.15 (10)	2.847 (5)	158 (10)
O21—H21A···O1^iv^	0.89 (4)	1.86 (4)	2.736 (5)	169 (6)
O21—H21B···O6^viii^	0.93 (9)	1.93 (9)	2.851 (5)	171 (8)
O22—H22A···O6^i^	0.89 (8)	2.49 (8)	3.127 (5)	128 (6)
O22—H22A···O10^i^	0.89 (8)	2.35 (8)	3.116 (5)	143 (7)
O22—H22B···O23^vi^	0.84 (4)	2.12 (7)	2.884 (7)	150 (6)
O23—H23A···O5^viii^	0.76 (10)	1.99 (10)	2.736 (6)	170 (12)
O23—H23B···O24^vii^	0.71 (9)	2.30 (11)	2.926 (6)	149 (16)
O24—H24A···O15^iii^	0.87 (15)	2.02 (15)	2.882 (5)	170 (15)
O24—H24B···O22	0.9 (2)	1.9 (2)	2.764 (6)	161
O25—H25A···O24	0.92 (11)	2.52 (11)	3.137 (7)	124 (10)
O25—H25B···O1^iii^	0.91 (10)	2.43 (12)	3.120 (7)	133 (12)
O25—H25B···O17^iii^	0.91 (10)	2.32 (13)	3.034 (7)	136 (15)
C5—H5···O16^ii^	0.93	2.46	3.380 (5)	170
C11—H11···O9^ix^	0.93	2.40	3.193 (6)	143
C24—H24···O8^iv^	0.93	2.45	3.172 (5)	135
C29—H29···O5^ix^	0.93	2.54	3.350 (6)	146
C30—H30···O7	0.93	2.28	3.001 (5)	134
C31—H31···O25^vii^	0.93	2.45	3.225 (8)	141
C36—H36···O4^x^	0.93	2.34	3.178 (5)	150

Symmetry codes: (iii) *x*, *y*−1, *z*; (iv) −*x*+1, −*y*+2, −*z*+1; (v) −*x*+1, −*y*+2, −*z*; (vi) *x*, *y*, *z*−1; (vii) −*x*+1, −*y*+1, −*z*+1; (viii) *x*+1, *y*, *z*+1; (i) *x*+1, *y*, *z*; (ii) *x*−1, *y*, *z*; (ix) −*x*, −*y*+1, −*z*; (x) −*x*, −*y*+1, −*z*+1.
